# The Bona Fide Nurse

**Published:** 1919-12-13

**Authors:** 


### THE MATRONS' AND SISTERS' DEPARTMENT

### THE BONA FIDE NURSE.

It has always been well understood by those who have studied the matter of registration for nurses that no Bill to establish it could ever be acceptable to Parliament unless it took into consideration what may be called the vested interests of existing nurses. There are at the present moment a large number of women engaged in private and other nursing whose training does not come up to the standard towards which we press—*viz.*, three years in a general hospital of not less than 100 beds. Their training-school may not have come up to modern notions of efficiency, nor the term of training given in the institution have been of sufficient length to admit them to the register. There have been in fact countless impediments hitherto besetting women on the path to standardised efficiency But these women have at any rate started on their career and have presumably made a success of it, seeing that so large a number are earning their living by it in spite of the handicap with which they started. Would it be common justice officially to deny them the right to be registered as trained nurses, and thus expose them to the risk of losing their livelihood when no official guidance was given them at the outset? No body of Englishmen or English women would consider this fair play. And so among the admitted difficulties of forming a register in any profession this initial difficulty looms large, the admission to the register during its first two years of people whose training has in some particular or other been irregular if judged according to the new rules.

Nurses who have not studied the matter, and alas! we fear they are in the majority, are suddenly startled to hear of this inevitable consequence of registration. They are offended at the bare idea of being classed with some who have had poorer opportunities than themselves of becoming proficient, and they would like to keep the register for nurses who can come up to the standard, say, of the College of Nursing, and let the rest get along as best they can, as in fact they have done hitherto. But they forget that the whole outlook will be changed by the setting of a register. Its object is to make the public suspicious of every one whose name is not entered on it, and it would do a great deal of harm to the fully-trained nurse if it were put about that the unregistered were just as good as the others, "less arrogant" (one hears them say), and possibly to be had at lower terms. The register must unite the profession, not leave outside it a great body of earnest workers penalised for no fault of their own.

All the same, we need not lightly assume that every woman who has lent a hand in her neighbourhood as sick attendant or has been a probationer at a cottage hospital, or has put in a few months' training at a fever institution, or has been privileged to assist in an auxiliary hospital as a V.A.D., will be automatically entitled to be registered as a trained nurse during the period of grace. The wording of the Bill is very guarded and affords the trained nurse and the public quite adequate protection.

Among the purposes for which the Council is empowered to make rules is that of "enabling persons, who within a period of two years after the date on which the rules to be made under the provisions of this paragraph first come into operation, make an application in that behalf, to be admitted to the register on producing evidence to the satisfaction of the Council that they are of good character, are of the prescribed age, are persons who were for at least three years before the first day of November, 1919, *bonâ fide* engaged in practice as nurses in attendance on the sick under conditions which appear to the Council to be satisfactory for the purposes of this provision, and *have adequate knowledge and experience of the nursing of the sick.*"

The Council, which will have to decide whether the applicants have had "adequate knowledge and experience of the nursing of the sick," is to be composed mainly of nurses, among whom there will presumably be matrons capable of forming a good judgment. Cannot this weighty matter be safely left in their hands? In the corresponding matter of the midwives, there was so great a scarcity of trained women that it was necessary to let any applicant on to the register if she had not got into trouble either from the point of view of the parson or of the doctor. The case is entirely different with nursing. Owing to the wise delay that has taken place in setting up the Nurses' Register, adequately trained nurses are by now in an overwhelming majority, and the public need not be left to the ministrations of uninstructed persons. Hard-and-fast rules cannot be applied at the outset of registration. What fairer plan could be devised than that of leaving each case to be judged on its own merits by a jury composed of persons whose business it is to gauge the essentials of a nurse's training?

